# Transcriptional and structural impact of TATA-initiation site spacing in mammalian core promoters

**DOI:** 10.1186/gb-2006-7-8-r78

**Published:** 2006-08-17

**Authors:** Jasmina Ponjavic, Boris Lenhard, Chikatoshi Kai, Jun Kawai, Piero Carninci, Yoshihide Hayashizaki, Albin Sandelin

**Affiliations:** 1 Genome Exploration Research Group (Genome Network Project Core Group), RIKEN Genomic Sciences Center (GSC), RIKEN Yokohama Institute, Yokohama, Kanagawa, 230-0045, Japan; 2 MRC Functional Genetics Unit, Department of Physiology, Anatomy and Genetics, University of Oxford, Oxford OX1 3QX, UK; 3 Computational Biology Unit, Bergen Center for Computational Science, University of Bergen, HIB, Thormøhlensgate 55, N-5008 Bergen, Norway; 4 Genome Science Laboratory, Discovery and Research Institute, RIKEN Wako Institute, Wako, Saitama, 351-0198, Japan

## Abstract

**Background:**

The TATA box, one of the most well studied core promoter elements, is associated with induced, context-specific expression. The lack of precise transcription start site (TSS) locations linked with expression information has impeded genome-wide characterization of the interaction between TATA and the pre-initiation complex.

**Results:**

Using a comprehensive set of 5.66 × 10^6 ^sequenced 5' cDNA ends from diverse tissues mapped to the mouse genome, we found that the TATA-TSS distance is correlated with the tissue specificity of the downstream transcript. To achieve tissue-specific regulation, the TATA box position relative to the TSS is constrained to a narrow window (-32 to -29), where positions -31 and -30 are the optimal positions for achieving high tissue specificity. Slightly larger spacings can be accommodated only when there is no optimally spaced initiation signal; in contrast, the TATA box like motifs found downstream of position -28 are generally nonfunctional. The strength of the TATA binding protein-DNA interaction plays a subordinate role to spacing in terms of tissue specificity. Furthermore, promoters with different TATA-TSS spacings have distinct features in terms of consensus sequence around the initiation site and distribution of alternative TSSs. Unexpectedly, promoters that have two dominant, consecutive TSSs are TATA depleted and have a novel GGG initiation site consensus.

**Conclusion:**

In this report we present the most comprehensive characterization of TATA-TSS spacing and functionality to date. The coupling of spacing to tissue specificity at the transcriptome level provides important clues as to the function of core promoters and the choice of TSS by the pre-initiation complex.

## Abstract 2

Investigations of the spacing between TATA box and transcription start site in mouse core promoters reveals a coupling of spacing to tissue specificity.

## Background

Elucidation of the mechanisms that govern the regulation of genes at the transcriptional level remains one of the most important challenges in biology. Transcriptional regulation is achieved by a combination of cellular events, including binding of *cis*-regulatory elements to transcription factor binding sites (TFBSs), chromatin structure modification, and the assembly of the pre-initiation complex (PIC) at transcription start sites (TSSs) [1].

Presently, we have a reasonable understanding of components used in the transcription initiation process but only limited insight into the mechanisms of the cognate elements [2-6]. The generally accepted model for transcriptional initiation by core promoter elements is centered on the complexes formed by TATA box binding protein (TBP) with RNA polymerases and associated factors [1]. It is a common textbook-inflicted misconception that 'typical' RNA polymerase II eukaryotic core promoters have a TATA box guiding the PIC. Recent evidence [7,8] provided genome-wide confirmation of the existence of at least two distinct modes of transcription initiation: CpG-island based, TATA independent initiation with multiple TSSs; and TATA dependent initiation, in which TSSs are concentrated on one or few consecutive genome positions (called single peak [SP] promoters). SP promoters and, by association, TATA-driven promoters are strongly associated with genes with tissue-specific and/or context-specific expression [8]. This is in agreement with recent large-scale statistical studies that confirmed the previously anecdotal correlation between CpG island promoters and housekeeping genes on one hand, and TATA box promoter and tissue-specific genes on the other [9]. The fact that TATA box promoters evolve more slowly than other types of promoters [10] implies that changes in such promoters are less tolerated and that this type of mechanism is more ancient than the more plastic promoters with many TSSs [8], in which evolutionary events can include evolutionary turnover [11].

In TATA driven promoters, the primary role of the TATA box is to anchor the PIC. In higher eukaryotes, this process sterically constrains the selection of transcription initiation sites, but TATA-TSS distance can vary slightly. The exact mechanism of start site selection, and therefore the TATA-TSS distance, remains unknown [3,12].

Because TATA boxes are highly overrepresented in promoters where the TSSs are concentrated in one or few consecutive genome positions, the TATA box location relative to the TSS is likely to have an impact on the efficiency of inducible expression. The unavailability of precise TSS locations has limited the study of the TATA-TSS spacing to a handful of promoters [13-18]. These studies indicated that the TATA box is functionally linked to the determination of the initiation site, and that TATA-TSS spacing affects the efficiency of transcriptional initiation.

It is evident that inducible expression is not solely orchestrated by events at the core promoter, but is also subject to long-range *cis*-regulatory element interactions [1] as well as cellular events on a larger scale, including epigenetic control of chromatin superstructure [19]. Nonetheless, core promoter elements have been confirmed as important determinants for transcriptional specificity [3], and our goal in this work is to determine the constraints imposed on such determinants.

We recently showed that the FANTOM cap analysis of gene expression (CAGE) data allow us, for the first time, to analyze simultaneously the precise locations of TSSs and the spatiotemporal expression patterns of the corresponding transcripts [8]. This permits detailed analysis of constraints imposed on TATA driven promoters for regulating inducible expression. Here, we show that, in TATA-driven promoters, the TATA-TSS spacing affects the transcriptional specificity of the downstream transcript. We then proceed to show that different TATA-TSS spacings affect a number of core promoter features, including the consensus sequence of the -3 to +1 region and the distribution of alternative TSS. Finally, we show that the overall TSS distribution within SP class promoters is indicative of tissue specificity as well as TATA box and initiation site properties.

## Results

### CAGE data and promoter classifications

CAGE [20,21] enables genome-wide localization of TSSs by rapid large-scale sequencing of 5' ends of mRNAs. The data structure and content of the CAGE data repository were described by Carninci and coworkers [8]. CAGE tags consist of sequenced 20-21 base pair (bp) long, 5' ends of full-length cDNAs that have been mapped to the corresponding (mouse or human) genome. Protocols for CAGE were described by Kodzius and colleagues [21]. Overlapping tags on the same strand form a tag cluster (TC) [8]. A TC and its surrounding genomic sequence can be considered a core promoter and is the basic unit used in this work.

A wide variety of RNA libraries (209) and tissues (23) was used for CAGE sequencing in mouse. Because all CAGE tags originate from defined RNA libraries isolated from specific tissues, for each TSS detected by CAGE the distribution of source libraries and tissues is also available. There are multiple lines of evidence for the high reliability and nucleotide-level resolution of CAGE tags, as discussed in detail in the supplementary material presented by Carninci and coworkers [8].

As discussed above, we previously discovered that promoters where the vast majority of TSSs are constrained to one to four consecutive nucleotides are enriched for TATA boxes and are associated with tissue-specific expression [8]. In the present study, in order to avoid ambiguous estimation of TATA-TSS distances, we analyzed promoters that have a single dominant peak located at a single nucleotide position (see Materials and methods, below). We shall refer to this type of promoters as 'single-TSS' promoters. For clarity, they are a subset of the SP promoter class, as defined by Carninci and coworkers [8].

**Figure 1:**
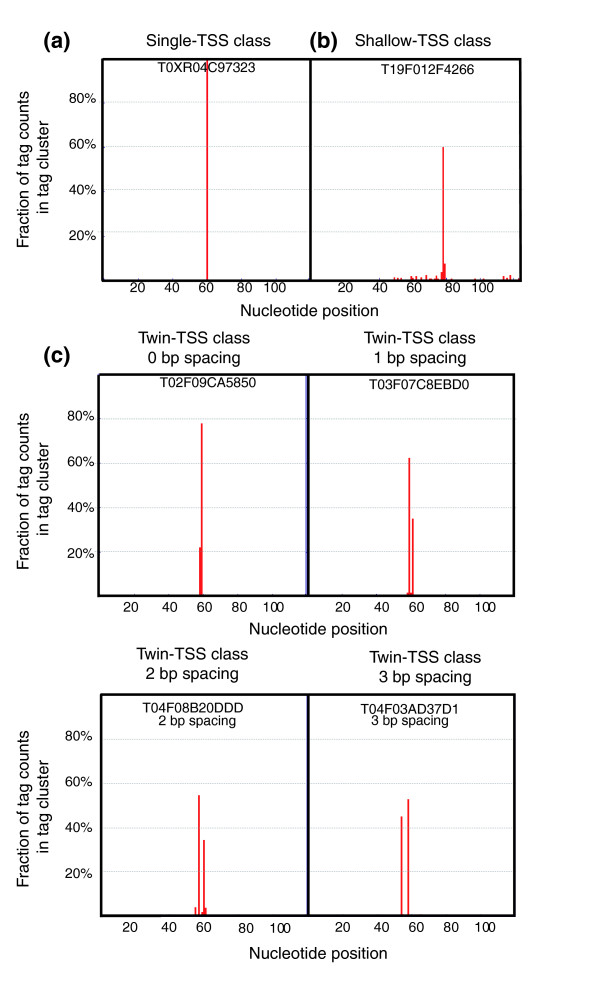
Representative examples of subclasses of SP promoters. Histograms show the fraction of tags that map into the 120 bp region centered on the TC. TC identifiers are shown above each histogram. Three subclasses of the SP TCs defined by Carninci and coworkers [8] were analyzed: **(a) **single-TSS promoters having a single well defined TSS; **(b) **shallow-TSS promoters, which is the subset of single TSS promoters that have one sharp peak surrounded by multiple weakly defined TSSs; and **(c) **twin-TSS classed promoters, which are characterized by two closely located, well defined TSSs, and in turn can be classified by the number of base pairs in between them (0-3 bp spacing). bp, base pair; SP, single peak; TC, tag cluster; TSS, transcription start site.

In the final part of the Results section, below, we also analyzed the properties of two related promoter classes: the subset of the single-TSS promoters that have a dominant single peak in combination with a uniform distribution of CAGE tags stretching over 50 bp; and the distinct set of promoters having two closely located dominant peaks (see Materials and methods, below, for exact definitions and Figure 1 for representative examples).

### Measuring tissue specificity using CAGE expression data

To assess the specificity of the expression of the downstream gene, we compared the tissue distribution of the CAGE tags within the TC with the tissue distribution of all CAGE tags, by computing the relative entropy (the Kullback-Leibler distance) [22,23] between the two distributions (see Materials and methods, below).

The concept of relative entropy has been applied to diverse computational biology problems, including sequence conservation [24], single nucleotide polymorphism selection [25], binding site predictions [26], and gene expression analysis [9,23,27-31]. Yan and coworkers [31] recently showed that relative entropy can distinguish differentially expressed genes better than other popular methods, such as *t*-tests, whereas Kasturi and coworkers [28] showed that clustering of gene expression using relative entropy was superior to Pearson correlation. In particular, Shannon entropy has been used in a number of studies to analyze transcriptional specificity based on cDNA and expressed sequence tag (EST) libraries [9,30]. Stekel and coworkers [30] presented a detailed study of statistical properties of related metrics in this context, whereas Schug and colleagues [9] showed that entropy-based metrics are useful for classifying expression profiles in GNF Gene Expression Atlas [32] and EST libraries as source datasets.

To demonstrate that relative entropy in combination with the CAGE data correlates with tissue-specific expression, we collected three sets of genes expected to be ubiquitously expressed: a set of 263 housekeeping genes from the HuGEIndex database (identified from microarray experiments) [33]; 14 genes of the citric acid cycle; and 23 genes of the ubiquitin-mediated proteolysis pathway, as annotated in the KEGG database [34]. We then collected six gene sets identified as tissue-specific using diverse approaches: 17 whole-brain specific genes (based on microarray expression profiles) [35,36]; 10 heart-specific genes (based on statistical over-representation in EST libraries) [37]; nine testis-specific genes (based on microarray expression profiles) [35,38]; 66 liver-specific genes; 12 lung-specific genes; and 20 cerebellum-specific genes, all from the GNF Gene Expression Atlas [32]. We then calculated the tissue specificity for each gene in the sets using relative entropy based on CAGE tags as well as on an independent dataset of EST cluster expression profiles within UniGene [39] (see Materials and methods, below).

**Figure 2:**
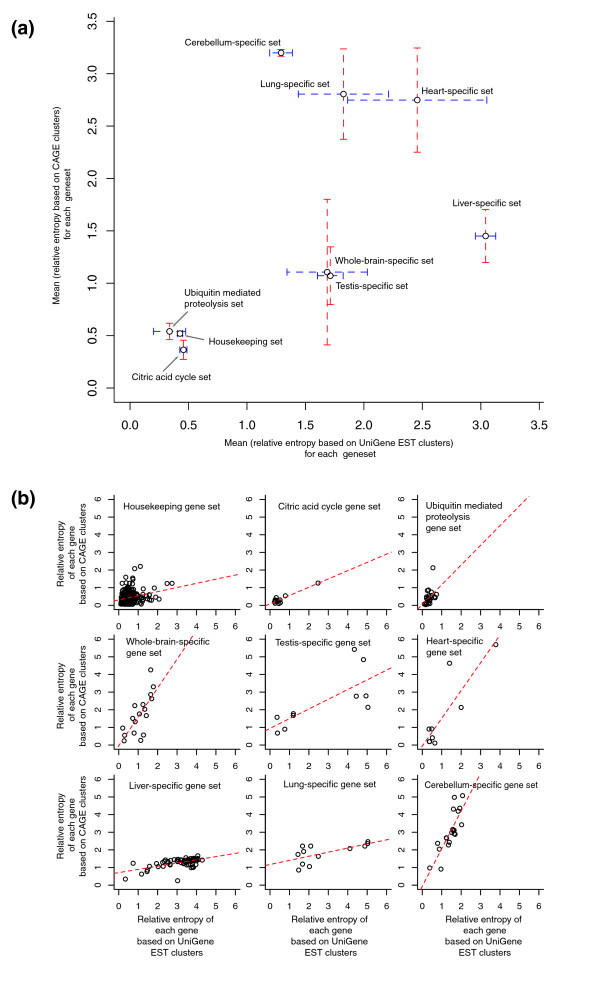
Tissue specificity measured by relative entropy. **(a) **Tissue specificity correlation between EST and CAGE data sources, measured as the mean relative entropy in each of the nine gene sets. Standard error bars for CAGE (red) and EST (blue) are shown. The plots of the six tissue-specific sets are distinct from the three ubiquitously expressed sets. **(b) **Tissue specificity correlation between EST and CAGE data sources, using the tissue specificity (relative entropy) of individual genes in each set. Spearman correlation coefficients and associated *P *values rejecting the null hypothesis (no correlation) are shown in Table 1. CAGE, cap analysis of gene expression; EST, expressed sequence tag.

The estimates of tissue specificity by CAGE and ESTs in almost all cases are significantly correlated when assessing single genes (Table 1 and Figure 2b). Because CAGE and ESTs are different and independent data sources, this is an additional piece of evidence that supports the validity and resolution of CAGE data, and supports relative entropy as a measure of tissue specificity. It is also immediately obvious that relative entropy separates the ubiquitous genes from tissue-specific genes when assessing the mean tissue specificity for each gene set (Figure 2a).

High relative entropy signifies great discrepancy between the TC tissue distribution and the background tissue distribution, and therefore temporally or spatially constrained expression of the corresponding gene, whereas two identical distributions will have a relative entropy value of zero. In this report we refer to the relative entropy measurement between the sample and expected distribution as the 'tissue specificity' or 'transcriptional specificity'.

### TATA-TSS spacing is associated with transcriptional specificity in vertebrates

A previous, basic descriptive analysis of the distribution of TATA-TSS spacing established that the most common spacings are 30 and 31 bp and that the great majority of TATA-driven promoters have a distance of 27-34 bp between TATA and the TSS [8] in mouse. Because our goal in this work was to elucidate whether there is a link between transcriptional specificity and TATA-TSS spacing, we sought both to increase the number of promoters analyzed and to focus on cases in which the TATA-TSS distance is unambiguous. Therefore, we applied a more conservative detection procedure to a larger amount of core promoters where the absolute majority of TSSs were concentrated on a single nucleotide position (the single-TSS class of TCs [see Materials and methods, below]). Only promoters with at least one predicted TATA box with a score greater than 75% within the -40 to -19 bp region relative to the dominant start site were used for subsequent analyses. This resulted in 784 single-TSS promoters used for the subsequent analysis.

Initially, we focused on the most prominent TATA box found in each single-TSS promoter (the highest scoring predicted) [6,40] TATA box location. We then measured the spacing between the first T in the TATA box (as defined by Bucher [41]) and the highest CAGE tag peak found in the TC (for simplicity, we refer to this position as 'TSS'). The findings we present below are not dependent on these specific cutoffs; changes in score cutoff and/or application of cross-species filtering of the promoter sets give similar results (data not shown).

We assessed the impact of TATA-TSS spacing on overall tissue specificity by measuring the relative expression entropy of the TCs grouped by TATA-TSS distance, as described in Materials and methods (below). When discussing positions within the promoter, we use the word 'upstream' to mean in the 5' direction of a given location in the promoter, with respect to the strand of the produced transcript (in all relevant figures, this is equivalent to the left-hand side). Similarly, we use the word 'downstream' for locations 3' of a given position (right-hand side in figures).

**Figure 3:**
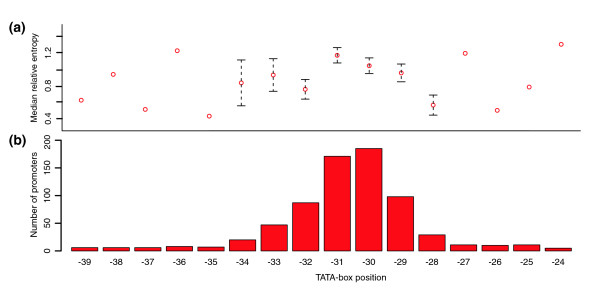
The spacing between TATA box and the dominant TSS is associated with transcriptional specificity. **(a) **Tissue specificity (measured as median relative entropy) for promoters with different TATA-TSS spacing. Positions with 20 counts or more are shown as red dots with standard error bars. **(b) **Histogram showing number of promoters with the TATA box located at a given position. In both plots, only the most prominent TATA box is considered in each promoter. Both representations indicate that most functional TATA boxes reside in a narrow 4 bp window from positions -32 to -29, dominated by positions -31 and -30. The rapid decrease in site counts and transcriptional specificity downstream of -29 suggests that 28 bp is the minimal TATA-TSS distance for TATA-driven initiation; it might also have functional properties distinct from more favorable spacings (see main text). bp, base pair; TSS, transcription start site.

When evaluating the results, it is important to consider both the median relative entropy (Figure 3a) and the count of promoters in each group (Figure 3b). A high promoter count in a given position implies a preferred TATA box usage of the position. Positions supported by 20 promoters or more have a distinct relative entropy distribution that reflects the corresponding site count distribution. Within this group, positions -31 and -30 have the greatest median tissue specificity, which is significantly higher than the preceding and following positions (-29 and -32: *P *= 4.3 × 10^-2 ^and *P *= 2.9 × 10^-2^, respectively; one-sided Wilcoxon test). They are also supported by the highest number of TATA boxes (Figure 3a,b). This implies that these two positions are the optimal TATA-TSS spacings for achieving high transcriptional specificity. TATA boxes downstream of -29 have lower specificity and radically lower counts; they are virtually never used in transcripts with high transcriptional specificity. It is therefore likely that 29 bp is the minimal spacing between TATA and TSS for effective transcription driven by the TATA box in a conventional manner (Figure 3a; see below for an analysis of atypical promoters with the TATA box located at -28). Upstream of -31, the three consecutive positions are viable as TATA box locations but are used less often; the tissue specificity and site counts diminish when moving from position -36 to -32. In large part, the varying median entropy values from positions -39 to -33 are due to low site counts in combination with a few extreme relative entropy values. This phenomenon might also be due to parallel usage of two TATA boxes, as discussed below.

**Figure 4:**
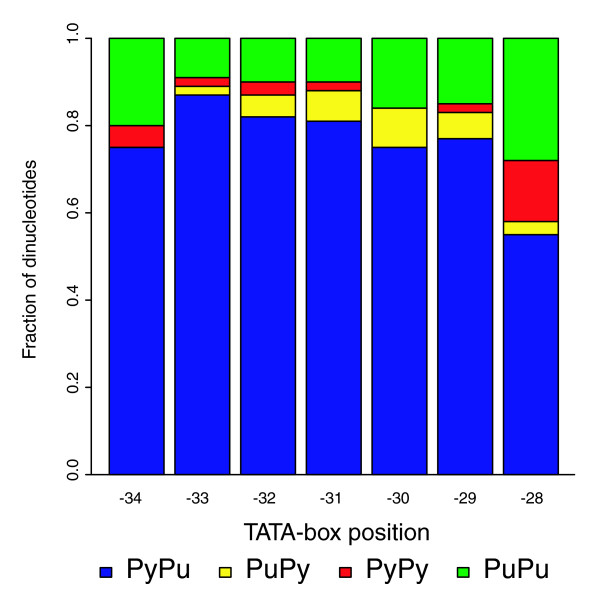
TATA-TSS spacing influences initiation site usage. Histogram showing the distribution of the four possible dinucleotides (PyPu, PyPy, PuPy, and PuPu) at the initiation site [-1, +1] for promoters with the TATA box located at each position in the -34 to -28 range. As described previously [8], initiation sites composed of PyPu dinucleotides are the most prominent, regardless of spacing. The dinucleotide distribution is significantly different for promoters where the TATA box starts at -28. Pu, purine; Py, pyrimidine.

**Figure 5:**
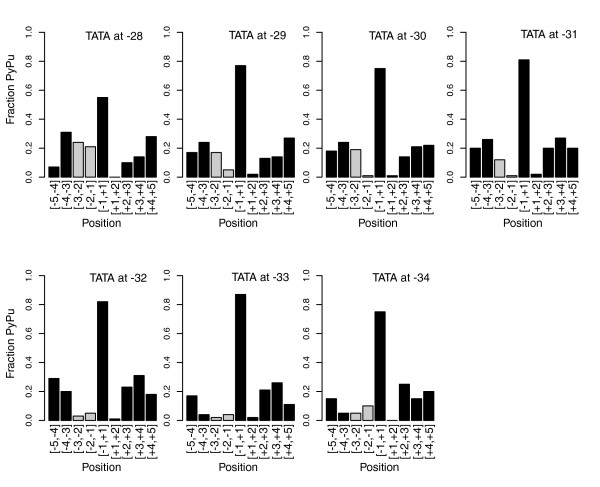
Extended TATA-TSS distances require unambiguous PyPu initiation sites. The fraction of PyPu dinucleotides in a sliding 2 bp wide window was calculated for each TATA spacing class in the [-5, +5] promoter region. Promoters with extended TATA-TSS distances (32-34 bp) are depleted of PyPu dinucleotides immediately upstream of the dominant TSS [-1,+1] (namely, [-2,-1] and [-3,-2]; fraction of PyPu dinucleotides shown as grey bars) and have a PyPu consensus at this site. Introduction of PyPu dinucleotides in this region would probably create new TSSs with a more favored distance to the TATA box. The PyPu distribution is largely symmetrical in promoters where the TATA box is located at position -31 to -29, indicating a possible intrinsic stretching mechanism within the PIC for selecting strong initiation sites located further away than the most favored distance (30 or 31 bp). bp, base pairs; PIC, pre-initiation complex; Pu, purine; Py, pyrimidine; TSS, transcription start site.

As previously shown, the preferred consensus for the initiation site is a pyrimidine-purine (PyPu) dinucleotide situated at position [-1, +1] relative to the TSS [8], corresponding to a weaker version of the previously defined Inr element [42]. Analysis of the preferred usage of initiation sites for different spacing classes provides additional insight (Figure 4). Promoters where the TATA box is located at -28 have a significantly different initiation site dinucleotide consensus compared with promoters that have other TATA box start locations (*P *= 3.1 × 10^-5^; χ^2^-test [see Materials and methods, below]). The initiation site dinucleotide distribution in promoters where the TATA box is located at -28 is also significantly different in pair-wise comparison versus positions -29 (*P *= 1.8 × 10^-2^), -30 (*P *= 1.6 × 10^-6^), -31 (*P *= 7.4 × 10^-4^), -32 (*P *= 1.6 × 10^-2^), and -33 (*P *= 1.5 × 10^-2^). In particular, the usage of the preferred PyPu dinucleotide is lower at position -28. This suggests that a different mechanism might govern this type of TATA-initiation site interaction. By comparing the use of PyPu dinucleotides in the region around the dominant TSS, we found that positions -34 to -32 are depleted of PyPu dinucleotides immediately upstream of the dominant TSS (Figure 5, grey bars), as compared with more favorable spacings. This is a strong indicator that introduction of PyPu sites in the depleted region would result in new TSSs with more favorable spacings. We show below that these atypical spacings are reflected in the overall promoter structure, both in terms of initiation site consensus and CAGE tag distribution.

### Correlation between TATA location and initiation signal

**Figure 6:**
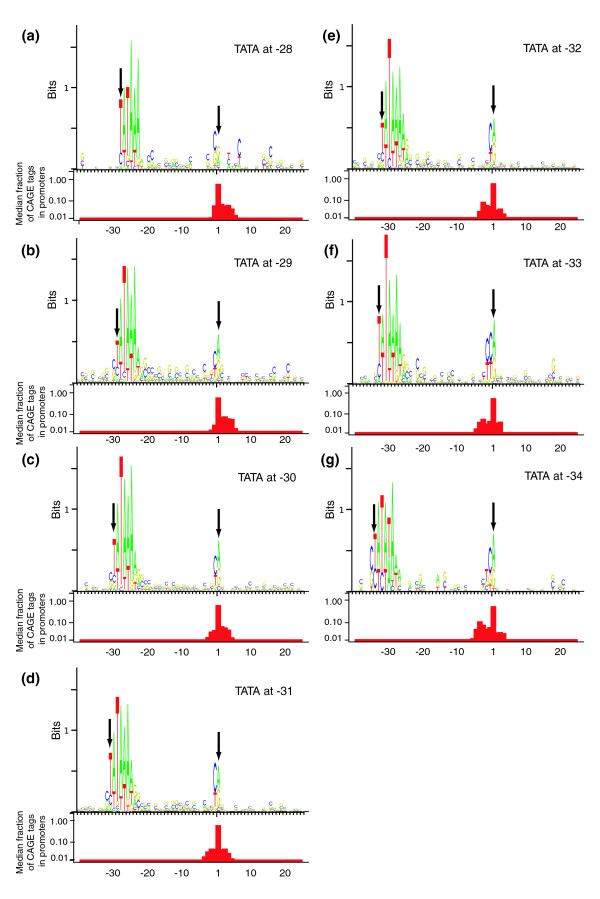
TATA-TSS spacing is correlated with promoter and initiation site characteristics. **(a-g) **Sequence logos [43] for promoters divided into spacing subclasses based on the location of the most prominent TATA box. CAGE tag distribution trends in each spacing subclasses are shown below each logo; specifically, the median fraction of CAGE tags within each promoter for each spacing class is plotted using a log-scaled y-axis (see Materials and methods). The locations of the dominant TSS and the TATA-box start are indicated with black arrows. Both the initiation site (positions -3 to +1) consensus and CAGE tag distributions differ between the different classes. Of particular interest is the extended initiation site motif for promoters located at -33 and -34, as well as the different consensus for promoters with TATA boxes located at -28. The CAGE tag distribution is skewed in a direction that is consistent with alternative start sites at a more favorable spacing (closer to position -30 or -31). CAGE, cap analysis of gene expression; TSS, transcription start site.

Because the different TATA-TSS spacings have different properties both in terms of tissue specificity and initiation signal, we investigated the core promoter regions (the -40 to +25 region relative to the dominant start site, defined as +1) of each subset using small sample corrected sequence logos [43,44] and normalized CAGE tag distributions (see Materials and methods, below; Figure 6).

Although small differences in TATA box consensus exist between spacing classes, the most important difference is in the properties of the sequence motif near the TSS; the initiation site consensus as well as the distribution of alternative TSSs are dependent on TATA-TSS spacing.

**Figure 7:**
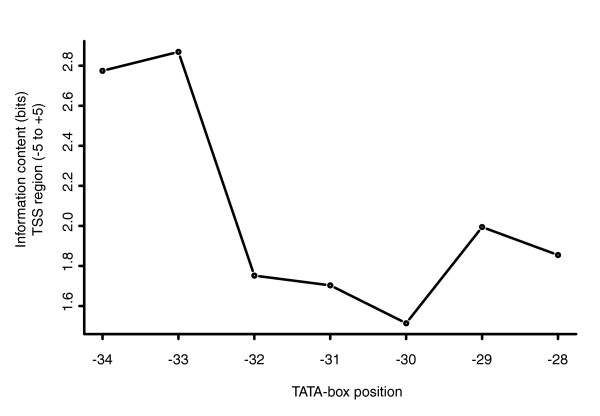
Non-optimal TATA-TSS spacing is compensated for by increased signal strength in the TSS region. The signal strength around the initiation site [-5,+5] (measured as information content in bits [45]) is lowest in promoters that have the most favored TATA-TSS spacings (30 and 31 bp). The signal strength is increased in promoters with a TATA-TSS spacing ranging from 32 to 34 bp. This increase is due to an extended initiation site motif, as shown in corresponding sequence logos in Figure 6. bp, base pairs; TSS, transcription start site. bp, base pairs; TSS, transcription start site.

For the four most favored spacings (TATA located at -29, -30, -31, or -32), the initiation site [-1, +1] is composed of a PyPu dinucleotide, which is consistent with work reported by Carninci and coworkers [8] and other studies [42]. The signal strength of the initiation signal (measured by information content [45,46] of the aligned region around the TSS [-5 to +5]) is slightly higher when the TATA box is located at position -32 compared with promoters with TATA boxes at the previous two positions (positions -30 and -31), and increases when the TATA box is positioned further upstream (positions -33 and -34; Figure 7).

**Figure 8:**
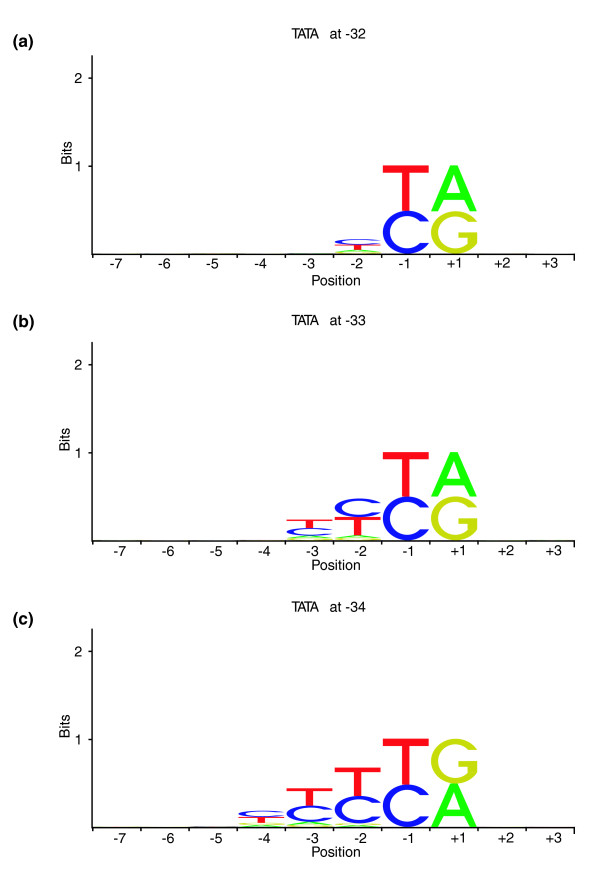
HMM simulations demonstrate increased signal strength as a result of PyPu depletion. Sequence logos resulting from sequence generation using an HMM incorporating rules for describing PyPu usage (see Materials and methods). Specifically, PyPu dinucleotides are not allowed in positions where they would introduce new initiation sites with more favorable TATA-TSS distances (-31, -32, and so on until the known spacing occurs). This results in an increase of Py nucleotides upstream of the TSS. bp, base pairs; HMM, Hidden Markov Model; Pu, purine; Py, pyrimidine; TSS, transcription start site.

When the TATA box is located at position -33 or -34, this increase is due to a gradually extended initiation site motif (Figure 6f,g). When the TATA box is located at position -33, the initiation site motif consists of a PyPu dinucleotide at [-1, +1], and a Py at -2. The reason for this is best explained by an example. Promoters with TATA boxes located at position -33 rarely have PyPu dinucleotides ending in positions -3 to -2 (Figure 5); consequently, the remaining alternatives are PyPy, PuPy, and PuPu dinucleotides. This would result in an over-representation of Py nucleotides in the second dinucleotide position. More importantly, once a Py nucleotide is chosen, each following nucleotide must also be a Py (until the true initiation site is reached) because the alternative would create a PyPu initiation site at a more favorable position. To determine whether we can reproduce these observations by simulation of the described constraints, we constructed a rule-based hidden Markov model (HMM) [47,48] that generated promoters where PyPu dinucleotides were not allowed in the region upstream of the TSS (see Materials and methods, below). Using the HMM, we generated three sets of 1000 promoters corresponding to TATA-TSS spacings of 32-34 bp (Figure 8). The generated promoters exhibit a gradual increase in Py nucleotides immediately upstream of the TSS. This is consistent with the observed promoters having TATA boxes at -33, but less so for promoters with TATA boxes at -34 (Figure 6). The initiation motif of promoters with the TATA box at -34 is ambiguous: the [C|T] in position -1 is replaced by a [C|T|G], with two weaker [C|T|G] at positions -2 and -3. The weaker signal strength is possibly a consequence of the lower number of sites in combination with the small sample correction applied (see Materials and methods, below). As a result, we cannot claim with confidence that the -34 position differs in a fundamental way from position -33 except by being even less favorable and therefore rarely observed.

Given the findings above, we argue that the additional signal strength (Figures 6 and 7) found around the initiation site in promoters with extended TATA-TSS spacings is not due to the existence of shared PIC binding site motifs in these promoters, but is due to the absence of a PyPu transcription initiation site at a more favorable spacing (Figure 5). Because information content is a measure of constraints in selection of symbols (in this case nucleotides), negative selection against a subset of symbols will increase the information content.

Consistent with the previous initiation site analysis (Figure 4), promoters where the TATA box is located at -28 have a weaker initiation site with an SR consensus at [-1, +1] (Figure 6a). The atypical promoter structure together with the low tissue specificity suggests that the mechanism for TATA-driven transcription is different in promoters with this spacing type.

We checked for the possibility that the TATA boxes at -28 could actually represent *bona fide *TATA boxes at -30, which would render the TATA box at position -28 redundant. However, the logo summarizing TATA boxes detected at -28 shows no support for this explanation. Additionally, the promoter structure and differential use of initiation site sequences between the promoters with TATA boxes at -30 and -28 makes the proposition unlikely. If a majority of TATA boxes located at -28 had a functional (and preferentially used) TATA box at -30, then we would expect the initiation site distributions to be similar for both spacing classes. On the other hand, the logo representing the TATA boxes located at position -34 (Figure 6g) has a TATATAA consensus instead of the TATAAA seen in the other spacings. This clearly shows the potential for parallel usage of TATA boxes at positions -34 and -32 (using the first and second T in the TATATAA).

The CAGE tag distribution around the dominant start position also reflects the spacing classes (Figure 6). As expected, positions -31 and -30 have the smallest CAGE tag distribution skew (the number of CAGE tags at each side of the dominant start site is approximately equal). Interestingly, the CAGE tag distribution in promoters with TATA boxes at position -31 is close to perfectly symmetrical, whereas there is a small skew toward the larger spacings at promoters where the TATA box is located at -30 (Figure 6c,d). Promoters where the TATA box is located elsewhere exhibit a considerable skew, which is fully consistent with the location of the sites, because they are skewed in the direction of more favorable spacings; promoters where the TATA box is located at positions -28 and -29 have alternative TSSs located downstream of the main TSS. Conversely, promoters with the TATA box at -32, -33, and -34 have alternative TSSs upstream of the main TSS (Figure 6). In both cases, the effect of choosing the indicated alternative TSSs would be a TATA-TSS spacing of 30 or 31 bp. In the case of promoters where the TATA-box is located at -34, there is potential for usage of both alternative TSSs and alternative TATA-boxes, as discussed above.

### TBP binding strength has minor effects on transcriptional specificity compared to spacing

**Figure 9:**
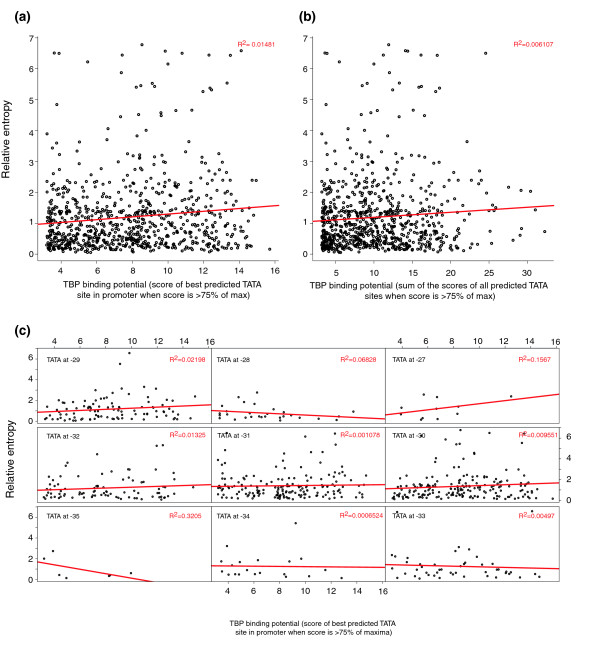
Exploration of the effects of TATA-TBP interaction strength on tissue specificity. We investigated possible dependencies between tissue specificity measured by relative entropy and three aspects of TATA-TBP interaction potential in the -40 to -19 region of each promoter: **(a) **the predicted TATA box with the highest score fulfilling the score threshold criteria defined in Materials and methods; **(b) **the sum of all predicted TATA boxes each fulfilling the specified score criteria; and **(c) **the predicted TATA box with the highest score fulfilling certain score threshold criteria, given TATA box location. For clarity, each plot in panel c corresponds to one type of TATA-TSS spacing, and can be considered a subset of the data points in panel a. The subdivision of the TATA-containing promoters into the different TATA-TSS spacing classes confers no additional support for a significant relation between TBP-TATA interaction strength and transcriptional specificity. In combination with panel a, this strongly suggests that TATA-TSS distance is more strongly linked to tissue specificity than the TATA-TBP interaction strength within TATA-driven core promoters. TBP, TATA box binding protein; TSS, transcription start site.

Having established that the spacing between TATA box and TSS is associated with transcriptional specificity, we investigated whether the strength of the TBP-TATA interaction has similar properties. The score of a predictive position weight matrix model is highly correlated with the strength of the protein-DNA interaction [40,49]. We only considered promoters with one or more TATA predictions having position weight matrix scores over the threshold of 75% [50], and focused first on the strongest TATA box. We could find no global correlation between binding strength and tissue specificity (*R*^2 ^= 1.5 × 10^-2^; Figure 9a), and neither could we establish any corresponding correlation when we subdivided the TATA boxes with respect to spacing (*R*^2 ^values from 6.5 × 10^-4 ^to 3.2 × 10^-1^, none of which is significant; Figure 9c).

Next, we investigated whether the existence of several, possibly overlapping *bona fide *TATA boxes in a single core promoter can influence the expression specificity, by analyzing the correlation between the sum of scores for all predicted TATA boxes, exceeding a 75% score threshold along the promoter (see Materials and methods, below), and their transcriptional specificity (Figure 9b). As above, we found no correlation (*R*^2 ^= 6.1 × 10^-3^). Finally, we repeated the same analysis with no score constraints in order to investigate whether the total binding potential for TBP along the promoter might have a significant influence (data not shown), but we found no correlation (*R*^2 ^= 8.1 × 10^-3^).

Taken together, these results imply that there exists a certain operational range of dissociation constant values for TBP-DNA interaction that is required for efficient TATA box guided transcription, but that there is no preferred strength of interaction within that range.

### Promoter shape modulates TATA-driven expression

Apart from the TATA-TSS distance, we found that the overall shape of the promoters within the SP class is indicative of transcriptional specificity and/or other promoter characteristics. We have focused on two 'borderline' subtypes of promoters found within the SP class set defined by Carninci and coworkers [8].

The first subtype includes promoters with a single peak in combination with a uniform distribution of CAGE tags stretching over 50 bp. We refer to these as 'shallow-TSS' promoters. This set is a subset of the single-TSS set analyzed above for TATA spacing properties.

The second subtype includes promoters with two dominant peaks with a spacing of 0-3 bp. We refer to these as 'twin-TSS' promoters. This set is disjoint from the single-TSS set.

Representative examples of tag clusters of the shallow-TSS and twin-TSS promoter subclasses are shown in Figure 1.

#### Shallow-TSS promoters are less effective for driving context-specific expression

We previously showed that promoters where the CAGE tags are distributed shallowly (the broad class [BR]) are associated with ubiquitously expressed genes and have high over-representation of CpG islands [8]. Therefore, it is not unreasonable that SP promoters with BR-like characteristics would be less suitable for directing specific expression. As described above, we tested the subset of 76 shallow-TSS promoters harboring TATA boxes against the remaining set of 708 single-TSS promoters harboring TATA boxes. The overall transcriptional selectivity of shallow-TSS promoter subset is lower (*P *= 4.0 × 10^-2^; one-tail Wilcoxon test), although the *P *value is marginally significant. Interestingly, this is also true if we only consider the dominant peak of the promoters in both sets (we ignore the flanking tags; *P *= 4.1 × 10^-2^; one-tail Wilcoxon test). Within a shallow-TSS promoter, the dominant peak generally has a higher transcriptional specificity than the flanking tags (*P *= 1.32 × 10^-4^; one-tail paired Wilcoxon test). Unexpectedly, the transcriptional specificity of the dominant peaks are highly correlated with that of the flanking tags (*P *< 2.2 × 10^-16^; two-sided Spearman rank correlation test), suggesting that the shape of these promoters cannot be explained by two overlaid tag distributions with different levels of tissue specificity.

#### Spacing between TSSs in twin-TSS promoters affects promoter structure

**Figure 10:**
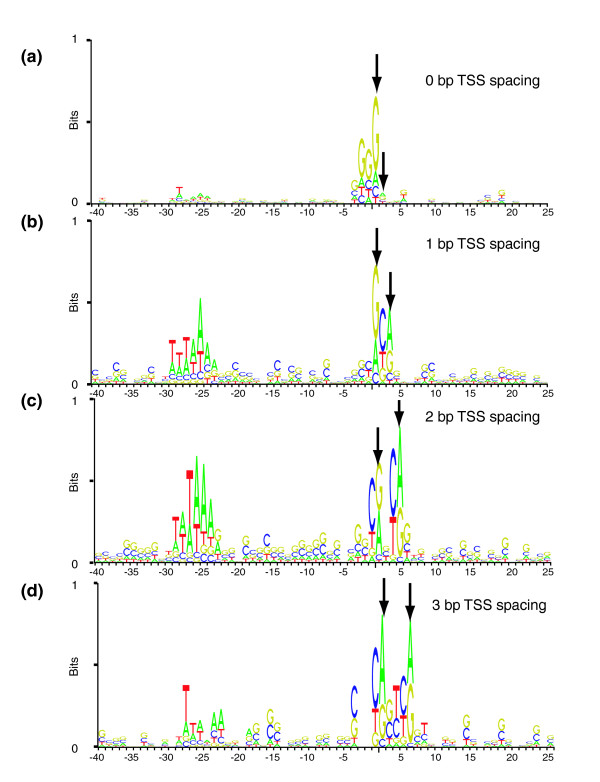
Exploration of SP-class promoters with twin-TSS. **(a-d) **Sequence logo representations of promoters with two close, dominant peaks separated by 0-3 bp. In contrast to previous sequence logos, we applied no constraint on TATA presence for promoter inclusion. Black arrows denote the location of the two dominant TSSs. The +1 position is arbitrarily defined as the position of the TSS located the furthest upstream. When there is no spacing between the peaks, promoters are depleted of TATA boxes. This type of promoter has an atypical initiation site consensus closely resembling that of transcripts in 3' untranslated region promoters [8]. More diverged peaks have a higher amount of TATA-like motifs around position -30 with respect to the most upstream peak. bp, base pairs; TSS, transcription start site.

As discussed above, the analysis of TATA-TSS spacing was focused on promoters where almost all TSSs are confined to a single nucleotide position. When preparing this set, we noticed a substantial number of promoters (465) that have two closely spaced dominant peaks (distance smaller than four nucleotides). We refer to this class of promoters as 'twin-TSS'. To investigate whether the TSS distribution can affect the promoter structure, we asked whether both peaks are associated with a TATA-like sequence about 30 bp upstream, or whether other mechanisms are employed, such as specific initiation site motifs. Regardless of TATA content, we subdivided the twin-TSS promoters with respect to the spacing between the two peaks, and constructed sequence logos by aligning each promoter centered on the peak located the furthest upstream (Figure 10). In the logos, we defined the +1 position to be the location of the first of the peaks. This definition is arbitrary and illustrates the disadvantage with the traditional annotation of the TSS as +1, in light of the CAGE data presented here and previously [8].

We found that promoters with a genomic spacing of 1-3 bp between the peaks have an unmistakable TATA consensus starting at around -30 and exhibit PyPu consensus initiation sites (Figure 10b-d). Conversely, promoters with two adjacent peaks (no spacing) have a significant under-representation of TATA boxes compared with the other twin-TSS promoters (*P *= 5.6 × 10^-6^; two-tailed Fisher's exact test [see Materials and methods, below]). These promoters also have a radically different signal near the initiation site: a GGG consensus, where the last G is located at position +1 (Figure 10a). Although the consensus is similar to the initiation site motif found previously in transcripts starting in 3' untranslated regions of protein encoding genes [8], we can at present only speculate on whether the mechanisms governing these types of promoters are similar.

We also investigated whether the transcriptional specificity of TATA-driven twin-TSS promoters is significantly different from that of single-TSS promoters. Intriguingly, the twin-TSS promoters might have a greater transcriptional specificity than the single-TSS promoters (*P *= 4.5 × 10^-2^; one-tail Wilcoxon test). However, because relative entropy values for the twin-TSS set are dominated by a few extreme outliers, it is unclear whether this observation holds in general. This implies that there are highly tissue-specific promoters that use two closely located TSSs, but it is unclear whether these are guided by two overlapping TATA boxes or by a mechanism in which the PIC chooses between the two comparably favorable TSSs (see Discussion, below).

## Discussion

### Determination of optimal TATA-TSS spacing

We have found that the spacing of the TATA-TSS is associated with tissue-specific expression (Figure 3). In particular, positions -31 and -30 are most strongly associated with context-specific transcription.

In comparison with the TATA-TSS spacing, the strength of TBP-TATA interaction does not appear to be correlated with the tissue specificity, only requiring that the interaction strength between TBP and a potential TATA box exceeds some threshold level.

The effects of TATA-TSS spacing on transcriptional specificity have been studied in depth within a few plant promoters. Zhu and coworkers [16] showed that, in *Oryza sativa*, the phenylalanine-lyase promoter activity *in vitro *was eliminated when a 6 bp element was either deleted from positions -21 to -16 or inserted between positions -18 and -19. This is entirely consistent with our more comprehensive study, because transferring the TATA box 6 bp upstream or downstream would take its starting locations outside the range of acceptable TSSs, as defined above. In a more detailed study of the developmentally important β-phaseolin gene promoter [17], multiple insertions and deletions were used to dissect the promoter function. Insertions between the TATA boxes and the initiation sites conferred either a significant decrease in transcription or creation of new TSS with a more favorable spacing (30 or 31 bp) relative to the TATA box, which is consistent with our analysis. Similarly, O'Shea-Greenfield and coworkers [15] showed that maximal expression in an *in vitro *system using human cell nuclear extracts was achieved when the TATA-TSS distance was 30 bp, and that when extending the distance from 30 to 35 or 40 nucleotides the start site was dislocated to a position 30 bp downstream of the TATA box.

Although our study shows the functional importance of the distance separating the TATA box and the TSS, the underlying mechanism that determines the start site selection is not fully understood, despite high-resolution X-ray structure determinations of the PIC and the polymerase II complex [5]. In TATA-driven promoters in higher eukaryotes, the TATA box functions as an anchor for the rest of the PIC, thus sterically focusing the selection of initiation sites to a limited range of positions. It is important to note that at present it is not fully understood whether the TATA-TSS spacing in itself contributes to changes in transcriptional specificity, or whether the observed spacings are consequences of other events, such as the mechanistic constraints imposed by the PIC and other *trans*-acting regulatory proteins.

A recent genome-wide survey of *Arabidopsis thaliana *core promoters [51] indicates that plant TATA box driven promoters probably share the spatial constraints presented herein. The authors estimated that the ideal TATA-TSS spacing in *A. thaliana *is 32 bp, but this analysis lacked the depth and resolution of TSS data that now are available for mouse [7,8]. The allowed TATA-box position distribution is similar to that of mouse, in which TATA boxes at positions closer than -29 are rarely observed, and larger distances are tolerated more often. The results in *A. thaliana *clearly show an immediate application for the insights we have presented in this study; the precise rules established here are valid across many eukaryotes, and can be applied for annotation of TATA-driven TSSs of those genomes in which the TSS data are not available or not precise enough.

### Promoter shape and initiation site consensus

As discussed, our results indicate that the TATA box must lie within a narrow 4 bp region (-32 to -29) in order to achieve high transcriptional specificity. When the TATA box is located within this region, the initiation site at [-1, +1] is dominated by a PyPu dinucleotide consensus. In the case of TATA motifs located upstream of -32, the PyPu consensus is retained but extended for TATA boxes at positions -33 and -34 (Figure 6f,g and Figure 7), which is due to an absence of PyPu initiation sites at more favorable distances upstream of the actual TSS (Figure 5). In these promoters there is also an evident skew in the CAGE tag distribution, indicating that if alternative minor start sites exist then they preferentially use more favorable spacings (closer to positions -30 and -31).

Our interpretation of these extended spacing classes can be divided into two different but not mutually exclusive hypotheses. First, because the TATA motif is more expanded and variable at these positions, there is a possibility that a weaker TATA box 2 bp downstream is used instead of the site indicated in our analysis. However, a consistent use of the downstream TATA box would not explain the high scoring TATA boxes at position -34 or -33, the skew of usage of minor initiation sites towards canonical spacing, or the depletion of PyPu dinucelotides at positions [-2, -3] (Figure 5), which is not present at other positions. A more likely explanation is the parallel use of both TATA boxes in the promoter. Only experimental follow up can resolve which of the putative sites is preferentially used.

A second, alternative explanation is that there is an intrinsic 'stretching' potential in the PIC anchored to the TATA box, resulting in the possibility of selecting TSS located further downstream when no suitable initiation site is present at the canonical distance. Promoters with a TATA box located at position -28 have a significantly different initiation site distribution in terms of PyPu (Figure 4). Because the PyPu initiation site is ambiguous in these promoters, it is reasonable to believe that the PIC stretching potential suggested above can accommodate extended but not decreased TATA-TSS distances. As in the case of more extended spacings, there is a skew in the CAGE distribution toward more canonical spacings.

These results suggest that the mechanism for TATA-TSS interaction by the PIC is comparable for promoters where the TATA box is located at positions -34 to -29. Conversely, the combination of atypical initiation sites and radically decreased transcriptional specificity for promoters where the TATA-box is located at position -28 suggests that this type of interaction is governed by at least partially different mechanisms.

### Correlation between CAGE distribution and TATA occurrence

In the concluding part of our analysis we looked at two related classes or promoters that depart from the 'ideal' single-peaked distributions. Our data indicate that the shallow-TSS class promoter might have a lower transcriptional specificity than the remaining single-peak class; this seems to be true also for the dominant TSS position.

The twin-TSS class promoters have a TATA box pattern when the spacing of the two dominant TSS peaks ranges from 1 to 3 bp. However, if the two TSS peaks have no spacing, then the promoters are TATA depleted and have a novel initiation site sequence motif (Figure 10).

The presented results demonstrate that the interdependence of TATA motifs and the associated TSSs reflect underlying promoter architecture and mechanisms.

## Conclusion

The underlying features of the CAGE data used in this study have enabled the discovery that TATA-TSS spacing is associated with the transcriptional specificity of the downstream transcript, the TSS distribution of the promoters, and initiation site motifs. Although our understanding of the functional mechanism that governs core promoters in general and the TATA-TSS interaction in particular is still limited, the results presented here will provide fertile ground for more detailed studies of core promoters. Our findings can be also used to resolve a substantial subset of ambiguities that arise from unreliable determination of TSSs, and will be an asset when annotating putative TATA boxes in uncharacterized promoters. The rules inferred for TATA boxes are directly applicable to the design of expression vectors in vertebrate systems, and suggest further directions in experimental investigation of transcriptional initiation from TATA-dependent promoters.

The combination of accurate, high-throughput TSS determination, systematic detection of *cis*-acting elements (for instance, ChIP2-chip [52,53]), and computational analysis offers a breadth of targets with a sufficient data depth to explore genome-wide principles. CAGE tag distributions reveal patterns of TSS usage in core promoters that will greatly advance our understanding of core promoter function and help to guide future promoter annotation and characterization experiments, both individual and genome wide.

## Materials and methods

### Experimental data sources

We used the FANTOM3 CAGE collection [7,8] for assessing TSSs in mouse (*Mus musculus*). The experimental procedure for production and mapping of CAGE tags to the genome Is described elsewhere [8,21]. The full set of 7,151,511 mapped CAGE tags was derived from 209 different RNA libraries and 23 tissues.

In our analysis we used a restricted set based on the 5,655,682 mapped tags originating from the 15 tissues, each containing at least 10,000 mapped tags. We removed tags from whole-body libraries, as well as macrophage libraries, because macrophages are present in almost all tissues and macrophage-specific genes have purine-rich proximal promoters that are not TATA associated [54]. The CAGE data are described by Kawaji and coworkers [55] and publicly available on the internet [56]. We consistently used the G-correction algorithm, as presented and used by Carninci and coworkers [8], for TSS locations.

### Promoter sets used in analyses

As in our previous study, CAGE TCs were used to define core promoter locations. Briefly, a CAGE TC consists of CAGE tags overlapping by at least 1 bp on the same strand [8]. Mouse TCs, containing at least 50 tags from the CAGE set defined above, were assigned a single peak shape (SP) if the distance between the 25 and 75 tag density percentile was less than 4 bp. A total of 2863 core promoters fulfilled this classification criterion and formed the initial set for selecting TATA-driven promoters; this is the same definition as was used by Carninci and coworkers [8], although that study used TCs with at least 100 tags. The reason for using the same initial definition was to make it possible to reflect our findings here with those made previously [8]. From this initial set we analyzed three subsets.

#### Twin-TSS promoters

A promoter was classed as twin-TSS if it fulfilled the following criteria: one of the neighboring TSSs (± 4 bp) relative to the highest TSS peak must contain at least 25% of CAGE tags of the highest TSS peak; and these two start site positions must contain more than 75% of the total CAGE tags within the TC. In total, 465 promoters were classed as twin-TSS, in which the lowest observed tag count for any of the two peaks was nine.

#### Single-TSS promoters

This is equivalent to the initial set of SP core promoters, excluding the twin-TSS promoters defined above. In total, 2398 promoters were classed as single-TSS, in which the lowest observed tag count of the main peak was 16.

#### Shallow-TSS promoters

This is a subset of the single-TSS promoters that fulfilled the following criteria: the TC must consist of at least 30 start site positions spanning a region greater than 50 bp; the sum of all tags within the TC, excluding those contained in the highest TSS peak, must be at least 100; and except for the dominant peak, each distinct start site must contain 20% or fewer of CAGE tags of the most dominant peak. In total, 185 promoters were classed as shallow-TSS.

### Determination of TATA box locations

We determined the occurrence of TATA boxes upstream 40 to 19 bp of the most dominant tag peak of these promoters by using the TATA model constructed by Bucher [41] deposited in the JASPAR database [57] and the TFBS Perl programming module [58] for predicting potential TBP binding sites. For clarity, the start of the TATA box was annotated as the first T of the TATA motif and the second position of Bucher's model.

For selecting likely TBP-binding sites we only accepted site predictions on the same strand as the transcript and exceeding a relative score threshold of 75%. For the different types of analysis in this work, we distinguished between three cases: we considered the best scoring TATA box prediction using the thresholds as above, the sum of all predicted sites with each site scoring greater than the defined threshold, or the sum of all predicted sites in the specified region without any relative score threshold constraint.

The vast majority of the analyses were made on the single-TSS promoter set. In total, 784 TCs in this set were assigned a predicted best scoring TATA box, whereas 2114 TCs in this set were considered when applying no relative score threshold criteria.

### Measuring transcriptional specificity using CAGE

Transcriptional specificity was measured by the relative entropy (the Kullback-Leibler distance) [22,59] of the tissue distribution of a sample TC with respect to the tissue distribution of all 5,655,682 CAGE tags:



where *k *is the number of different tissues (*n *= 15), *p *is the discrete probability distribution of tissues in the sample tag cluster, and *q *is the discrete probability distribution of tissues for all tags. The distance cannot be negative, and if *p *= *q *then the distance *d *will be 0.

### Measuring transcriptional specificity using ESTs

In a comparative study, we measured the transcriptional specificity based on EST clusters from the UniGene database; more specifically, the Mm.profiles file from the UniGene ftp repository [60] was used as a data source. It summarizes the expression profile of ESTs in each cluster from libraries with curated and controlled vocabulary tissue annotation, in which each cluster has at least 10 tags. Relative entropy was calculated using the equation given above, where *k *is the number of tissues, *p *is the discrete probability distribution of tissues in the sample EST cluster, and *q *is the discrete probability distribution of tissues for all ESTs.

### Extraction of tissue-specific genes from literature and Internet sources

The gene sets were taken from the supplementary material of each publication, except for the GNF-derived data, which were retrieved using the SymAtlas web tool [61]; we selected all mouse genes from the Mouse GeneAtlas U74A [32] set, which had an expression fold over 30 of the median using the web retrieval tool for liver and adipose separately. The same procedure was repeated for lung using a 25-fold threshold.

For all gene sets, we only included genes that were covered both by CAGE and EST data. To be able to compare EST clusters and CAGE TCs, we used the official mouse gene symbol names for linking purposes. In cases in which several alternative promoters existed in the CAGE database, we selected the TC with the largest number of tags. Within this analysis, we did not exclude macrophage and whole body libraries, because it was unreasonable to treat CAGE and EST sets differently. We also used a CAGE tag count threshold of 30 tags for TCs included in the analysis in order to be closer to the 10 EST threshold used in the UniGene cluster database.

### Analysis of differential initiation site distribution for promoters with the TATA box located at -28

We applied the χ^2 ^test for the frequency distribution, as implemented by Ihaka and Gentleman [62], of the four different dinucleotide classes (PyPu, PyPy, PuPy, and PuPu) at the initiation site [-1, +1] in order to determine whether the initiation site distribution from promoters in which the TATA box is located at -28 can be considered significantly different from the initiation site distribution from all promoters with the TATA box situated at position -34 to -29.

### Analysis of PyPu dinucleotide usage in the vicinity of observed TSS

We wished to assess the occurrence of PyPu dinucleotides immediately upstream and downstream of the TSS [-1, +1] in the different TATA-TSS spacing classes (-34 to -28). Using a 2 bp sliding window, we counted the PyPu dinucleotides in the region ± 5 bp of the TSS for each TATA-dependent promoter sequence, normalized by the number of promoters in each spacing class.

### Specific TATA-TSS sequence logos and corresponding CAGE tag distributions

We classified each TC in terms of the spacing between the best scoring TATA box prediction that fulfilled the selection criteria listed above, and the initiation site (the highest CAGE peak within the TC, for convenience referred to as 'TSS' and located at +1). We only considered TATA boxes in a restricted spacing interval from position -34 to -28, because the absolute majority of functional TATA boxes reside in this region (Figure 3b). We then extracted the -40 to +25 sequence region relative to the TSS and created a sequence logo [43] for each TATA-TSS spacing class using the TFBS programming modules [58]. Small sample correction was applied as described Schneider and coworkers [43,44] and implemented by Lenhard and Wasserman [58]. We measured the signal strength of the region surrounding the initiation site by calculating the total information content [46] of the -5 to +5 sequence region for each given spacing class.

In order to visualize the CAGE tag distributions in that region, the frequency distribution of CAGE tags was obtained for each TC (one bin per bp) and then normalized by its total number of CAGE tags within the TC. For each position in the logo, we calculated the median tag density from the array of vectors defined above.

### Hidden Markov model simulation for exploring signal strength effects of PyPu depletion

To investigate how the signal strength immediately upstream of the observed TSS is affected by depletion of PyPu dinucleotides, we constructed a simple HMM that generates sequences according to a set of rules. The rules are a simplification of the biologic reality, because the goal is just to explore the principal effects of PyPu depletion. The HMM generates a sequence corresponding to the regions surrounding the TSS (-7 to +3) from left to right using the following rule set: there must be a PyPu at [-1, +1]; when selecting a nucleotide at position *i *in the region immediately upstream of the [-1, +1] region, nucleotides [*i-1*, *i*] must not form a PyPu dinucleotide; and aside from these constraints, all nucleotides are considered equally likely for selection.

**Figure 11:**
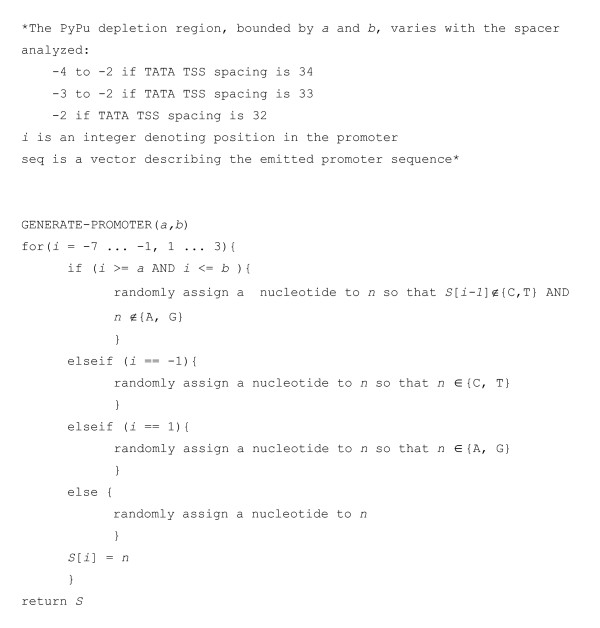
Pseudo-code corresponding to the Hidden Markov model simulation.

The length of the region subjected to PyPu depletion in the second rule is dependent on TATA location; if the TATA-box is located at -33, then PyPu dinucleotides introduced at positions -3 or -2 would correspond to new TSSs with more favorable spacings (-31 and -32, respectively). For promoters where TATA is located at -32 or -34 the region is [-2] and [-2, -3, -4], respectively. This process can also be expressed in pseudo-code, as shown in Figure 11.

### Software libraries

Unless otherwise indicated, we used the R language [62] for statistical analysis and graph visualization. We used the TFBS [58] library for promoter pattern analysis and sequence logo drawing, and the AT libraries (Engström P, Andersen M, Sandelin A, Fredman D, Lenhard B, unpublished data) for sequence handling and genome informatics.

## Figures and Tables

**Table 1:** Correlation of tissue specificities measured by relative entropy in CAGE and UniGene EST clusters

Gene set	EST versus CAGE: Spearman rank correlation coefficient	Spearman rank correlation *P *value	Number of genes
Whole brain specific	216	1.10 × 10^-3^	17
Testis specific	48	9.68 × 10^-2^	9
Heart specific	40	1.48 × 10^-2^	10
Liver specific	20,898	1.32 × 10^-6^	66
Lung specific	92	1.81 × 10^-2^	12
Cerebellum specific	186	<2.20 × 10^-16^	20
Citric acid cycle	318	2.90 × 10^-1^	14
Ubiquitin-mediated proteolysis pathway	886	5.94 × 10^-3^	23
Housekeeping genes	2,208,352	8.54 × 10^-6^	263
All sets combined	5,269,164	<2.20 × 10^-16^	434

Pair-wise correlations between tissue specificity values using CAGE and EST clusters was calculated as in the cor.test method in the R language [62], using Spearman correlation (two-sided test). CAGE, cap analysis of gene expression; EST, expressed sequence tag.
